# Automated Dashboards for the Identification of Pathogenic Circulating Tumor DNA Mutations in Longitudinal Blood Draws of Cancer Patients

**DOI:** 10.3390/mps6030046

**Published:** 2023-05-01

**Authors:** Aleksandr Udalov, Lexman Kumar, Anna N. Gaudette, Ran Zhang, Joao Salomao, Sanjay Saigal, Mehdi Nosrati, Sean D. McAllister, Pierre-Yves Desprez

**Affiliations:** 1 Graduate School of Management, UC Davis, 1 Shields Ave., Davis, CA 95616, USA; 2 California Pacific Medical Center, Research Institute, 475 Brannan St., San Francisco, CA 94107, USA

**Keywords:** liquid biopsy, metastasis, next-generation sequencing

## Abstract

The longitudinal monitoring of patient circulating tumor DNA (ctDNA) provides a powerful method for tracking the progression, remission, and recurrence of several types of cancer. Often, clinical and research approaches involve the manual review of individual liquid biopsy reports after sampling and genomic testing. Here, we describe a process developed to integrate techniques utilized in data science within a cancer research framework. Using data collection, an analysis that classifies genetic cancer mutations as pathogenic, and a patient matching methodology that identifies the same donor within all liquid biopsy reports, the manual work for research personnel is drastically reduced. Automated dashboards provide longitudinal views of patient data for research studies to investigate tumor progression and treatment efficacy via the identification of ctDNA variant allele frequencies over time.

## 1. Introduction

A major goal of precision medicine in cancer is to provide effective and specific therapies for patients by incorporating more biomarker-directed therapies [1]. To facilitate personalized treatment in cancer, studies pertaining to high-throughput drug screens of individual patient primary tumor cultures as well as the genomics landscape of their tumors are being evaluated, as part of the Cancer Avatar Project, at the California Pacific Medical Center Research Institute. We recently published our institutional experience of developing a liquid biopsy approach using circulating tumor DNA (ctDNA) analysis of plasma for personalized medicine for cancer patients. The focus of this study was on the hurdles encountered during the multistep process in order to benefit other investigators wishing to set up this type of study in their institution [2]. In this manuscript, we also describe some case reports using longitudinal samples, illustrating the potential advantages and rewards in performing ctDNA sequencing to monitor tumor burden or guide treatment for cancer patients.

Compared to traditional biopsies, liquid biopsies are more convenient, easily obtainable, and present minimal procedure risks to patients. ctDNA, as a part of circulating cell-free DNA (ccfDNA) in peripheral blood, contains gene mutations found in primary tumors, and the serial sampling of ctDNA can have diagnostic value and predict the response to treatment and the clinical outcome. Earlier studies have shown the potential power of this approach to monitor tumor burden in cancer patients [3,4]. So far, these results suggest the potential of ctDNA analysis in the monitoring of disease progression and treatment response in individual cancer patients [5,6,7]. 

Pharmacological studies focus on drug testing to estimate efficacy, while genomic analyses focus on the identification of pathogenic mutations in the patient [8]. The identification of specific pathogenic DNA mutations can drive the choice of the pharmacological agent used to treat a patient, and the presence of such mutations can be tracked over time using liquid biopsy tests. With over 600 liquid biopsy reports flowing into the laboratory computers, our process of identifying pathogenic mutations and organizing the reports by individual patients, which are appropriate for a research context independent of a clinical setting, had been a cumbersome manual process. 

Here, we describe an automated way of identifying pathogenic mutations from liquid biopsy reports and grouping them at a patient level. The method uses no patient identifiable markers and is essentially anonymous. We discuss the underlying theory of this process using the techniques in data science and provide a way to replicate it in any organizational setup. The focus of this paper is to provide a replicable, automated methodology to organize ctDNA information sourced from liquid biopsy and identify the patients to perform longitudinal analyses. 

## 2. Materials and Methods

### 2.1. Genetic Sequencing Method

#### 2.1.1. Circulating (Cell-Free) Tumor DNA Extraction 

Extraction was performed on 655 human plasma samples. Informed consent was obtained from all subjects involved in the study, which was conducted according to the guidelines of the Declaration of Helsinki and approved by the Institutional Review Board of Sutter Health (protocol code 2015.059-1 approved on 3 October 2022). Blood samples were collected in tubes (PAXGene blood tubes) with preservatives to increase shelf life and ccfDNA (which corresponds to DNA fragments shed by all cell types including cancer cells) was isolated using the QIAamp Circulating Nucleic Acid kit (Qiagen, Redwood City, CA, USA), and quantified using PicoGreen (Thermo Fisher Scientific, South San Francisco, CA, USA). 

#### 2.1.2. Next-Generation Sequencing

We selected the 56G Oncology Panel V2 from Swift Biosciences (Ann Arbor, MI), which contained 56 gene targets: ABL1, AKT1, ALK, APC, ATM, BRAF, CDH1, CDKN2A, CSF1R, CTNNB1, DDR2, DNMT3A, EGFR, ERBB2, ERBB4, EZH2, FBXW7, FGFR1, FGFR2, FGFR3, FLT3, FOXL2, GNA11, GNAQ, GNAS, HNF1A, HRAS, IDH1, IDH2, JAK2, JAK3, KDR, KIT, KRAS, MAP2K1, MET, MLH1, MPL, MSH6, NOTCH1, NPM1, NRAS, PDGFRA, PIK3CA, PTEN, PTPN11, RB1, RET, SMAD4, SMARCB1, SMO, SRC, STK11, TP53, TSC1, and VHL. MiSeq 2 × 151 base paired-end sequencing was performed to detect single-nucleotide variant (SNV) and insertion/deletion (indel) at 1% allelic frequency or higher in target regions with sufficient read coverage (at least 100×).

#### 2.1.3. Data Analysis

ccfDNA data obtained using the 56G Oncology Panel V2 was analyzed using Genialis Expressions (Accel-Amplicon analysis workflow, Genialis Inc., Boston, MA, USA). In brief, quality trimmed (Trimmomatic v.0.36) sequencing data was aligned to the human genome (GRCh37 assembly) using BWA MEM (v. 0.7.17-r1188). The aligned data were further processed by trimming primer sequences (Primerclip, Swift biosciences) and using GATK (v.3.6) tools (IndelRealigner and BaseRecalibrator) to prepare the analysis-ready BAM file. SNP/INDELs were named using LoFreq (v.2.1.3.1) and annotated using snpEff (v.4.3k).

### 2.2. Pathogenic Matching Approach

Reference data were obtained from the COSMIC (Catalogue of Somatic Mutations in Cancer) database that consolidates data from peer-reviewed publications and other genomic data screening sources in order to provide a comprehensive overview of cancerous genetic mutations [9]. This data source was filtered to reduce the number of variables to the gene name, amino acid mutation, type (pathogenic/neutral), and FATHMM (Functional Analysis through Hidden Markov Model) score. Duplicates were removed and the resulting data were uploaded into an SQL (Structured Query Language) database in order to automate the classification of liquid biopsy reports. COSMIC aggregates latest research on cancer-causing mutations and assigns a probability score for these mutations. When using this database, it is advisable to update the data source twice a year to identify new findings on novel pathogenic gene mutations.

Input Genialis files (or similar sequencing results with comparable structure) were uploaded into an internal database via Python script (an open-source object-oriented computer programming language). This programming code serves multiple functions beyond database creation and data load. Built-in functionality includes a check for whether the sample is already included in the database in order to prevent redundancy in data collection. The program identifies the level of pathogenicity, based on the gene and amino acid mutations within the sample and identical combinations in the COSMIC database, before consolidating data from both sources into a database for further analysis.

### 2.3. Patient Similarity Analysis

Liquid biopsy reports collected from the patients are anonymous. A major part of the genomic analyses involves tracking of pathogenic mutations that are detected from the bloodstream. With anonymity, there is a need to identify liquid biopsy reports that belong to the same patient. This could be accomplished by leveraging the fact that the fragments of ccfDNA detected from the bloodstream are unique to each human. We, therefore, attempted to quantify the similarity of liquid biopsy reports to identify if they would belong to the same patient. 

We leveraged the cosine similarity method from vector algebra [10]. The mathematics uses two objects—a scalar and a vector. Scalar is an object that can be represented as a single number; it has only magnitude. Since a vector has magnitude and a direction, it is plotted in an N-dimensional space. An example of a scalar is speed (which is simply a number representing magnitude), while the vector form is velocity (it has both magnitude and direction in a 3D space). Similarly, every liquid biopsy report can be represented as a vector with genes as dimensions and their allele frequency as the corresponding magnitude. The similarity of vectors can be quantified by measuring how close their projection is on one another. 

In order to increase the range of similarity scores, we added another layer before the similarity score calculation. We used k-means clustering method to separate the samples set into two groups to ease the computation process by reducing the number of samples that are compared to one another. This clustering method ensures that no two samples falling into the two different groups are similar to each other, but that each remains comparable to the ones within the group. Similarity scores were computed within each group, and any sample that had not seen a match based on the score was extracted from both groups. They were shuffled to increase the probability of obtaining the right match while reducing computation time overall.

### 2.4. Longitudinal Data Visualization

After the pathogenic matching and the patient similarity analysis were conducted, the final data were uploaded via the Python script into a database for integration with Tableau, a visualization platform [11]. This table includes indicators for germline mutations, defined as gene and mutation combinations with allele frequencies around either 50% or 100%. Additionally, a flag for allele frequencies < 1% was included in order to provide filtering options for visualization that retain only larger frequency pathogenic mutations to facilitate clinical analysis. Overall, all the necessary code to build this data platform can be found at the following website address: https://github.com/azurey0/cpmc-prac (accessed on 1 April 2023).

## 3. Results

The project’s initial objective was to improve research efficiency by automating the classification of pathogenic gene mutations presented in the liquid biopsy reports. As our understanding of the data structure improved, we uncovered a second objective: to expand the research by identifying the liquid biopsy samples that belonged to the same patient without the need for specific patient information. Lastly, we combined the gene classification with the patient matching to show how the pathogenic genes evolved in the same patient. To deliver the results, we used Tableau [11], in which the user can easily upload new samples and navigate and check information about the gene classification, patient matching, and longitudinal analyses. 

### 3.1. Pathogenic Mutation Matching

**Figure 1:**
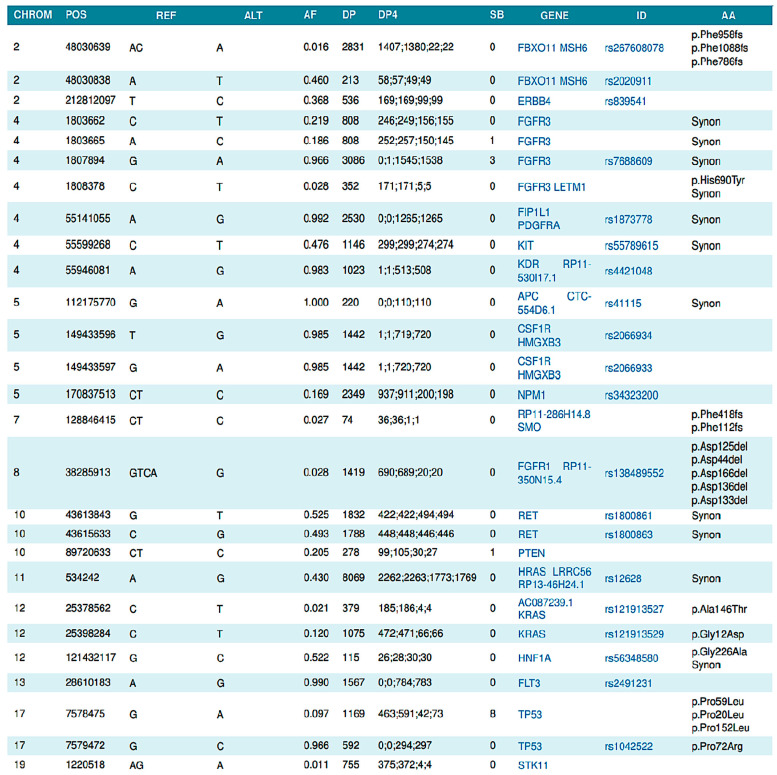
Example of report received from Genialis.

**Figure 2:**
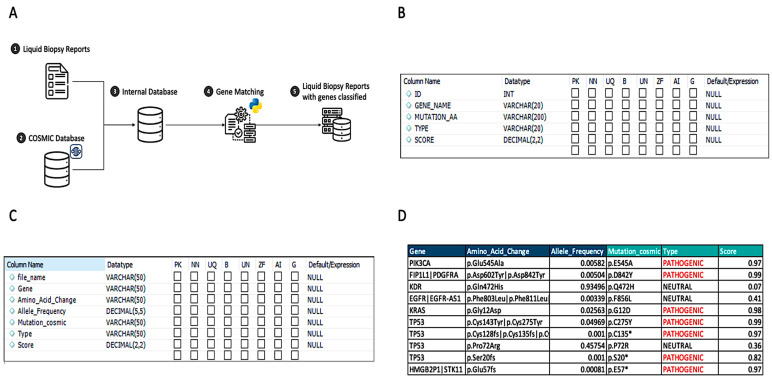
Pathogenic mutation matching. (**A**) Gene matching process workflow describing the process of merging two different datasets to create the final data. (**B**) MySQL Table Schema for COSMIC data source. (**C**) MySQL Table Schema for patient matching results. (**D**) Example of liquid biopsy report after gene matching process.

First, gene and mutation data points were mapped from Genialis standard forms (Figure 1) to the nomenclature utilized in the COSMIC database. Figure 2A provides an overview of the data processing flow for the matching methodology, Figure 2B provides the view of the database table for COSMIC data, and Figure 2C provides the patient matching results. The two sources were combined by matching the gene and amino acid changes, finding the associated FATHMM or pathogenicity score, and creating an output that combines both sources into a final consolidated data source (Figure 2D). 

During loading, the Python program presented in the Materials and Methods section identified the Amino Acid Change column (column “AA” in Figure 1) and the Gene column (column “GENE” in Figure 1) of the input file. The program then translated the AA column into the nomenclature used in the COSMIC database (Figure 2B) and then matched the same columns in the COSMIC database and determined whether COSMIC classified each gene and mutation combination as Pathogenic or Neutral. The Python script found COSMIC’s associated FATHMM score, which is a measure of pathogenicity severity (1 being the highest and 0.8 being the cut off for pathogenic mutations) and then loaded the new sample data into the Patient Matching database (Figure 2C). Additionally, the code checked whether the number of rows and a ratio of the number of rows to the total number of genes in the sample report were within normal ranges. Files with less than 32 total gene and mutation combinations, or that had a ratio of mutations to genes greater than 0.25, were flagged as potentially having an issue with the genetic sequencing for research and clinical consideration. This issue was also notable in the patient similarity analysis below.

A manual review of more than 600 unique liquid biopsy samples compared to this automated methodology successfully identified all previously known pathogenic mutations for the samples at a benchmark of a FATHMM score greater than 0.8 (Figure 2D). Additionally, several mutations were identified as pathogenic that had not been isolated during the manual review. The subsequent quality check indicated that the programmatic method of classifying genetic mutations was sufficient. In combination with the benefit of liquid biopsy results as a non-invasive alternative to identifying cancer patients that have ctDNA detectable in the blood, this program results in a greater speed for obtaining final liquid biopsy analysis results for research and, potentially, clinical use.

### 3.2. Patient Matching

**Figure 3:**
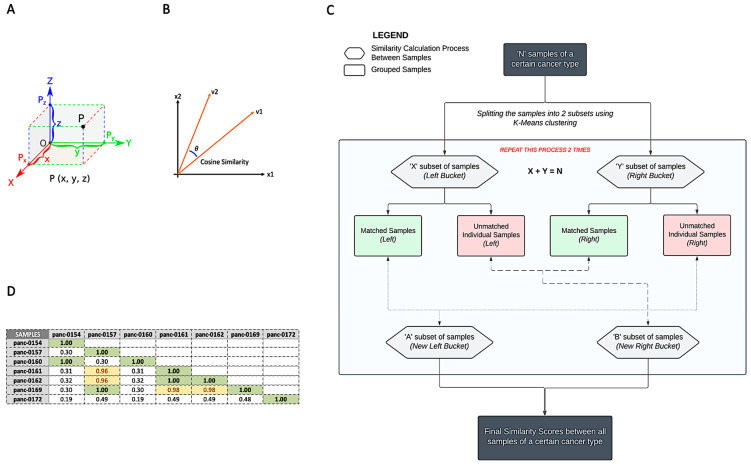
Patient matching. (**A**) Mathematical representation of a point P in cartesian three-dimensional coordinates that describes the vector notation. (**B**) Cosine similarity of two vectors: a mathematical measure to find the similarity of two vectors by the angle made between them. The smaller the angle, ‘theta’, the higher the similarity. (**C**) Similarity score calculation process in the form of a flowchart. (**D**) Similarity scores of 7 liquid biopsy reports—sample results are based on the described methodology (in yellow, score range between 0.95 and 0.98).

As presented in the Materials and Methods section, we utilized a cosine similarity algorithm [10] to perform the patient matching required for the longitudinal monitoring of the liquid phase biopsy results. A simplified version of a 3D vector is shown in Figure 3A, in which the line joining OP is a vector pointing towards P. The x, y, and z components are the dimensions of the vector while P_x_, P_y_, and P_z_ are their corresponding magnitudes. Extrapolating this to the liquid biopsy reports, we represented each row of Figure 1 as a vector with genes as the dimensions and their allele frequency as the corresponding magnitude. 

In Figure 3B, we present two vectors with an angle, ‘theta’, between them. Assuming the vectors are of an equal length, when ‘theta’ is zero, they are most similar (similarity of 1), and when the angle is 90 degrees, they are least similar (similarity of 0). This method involves the computation of the cosine projection [10]. We used this technique to compute the similarity scores of any two liquid biopsy reports. Using the panel that comprised 56 genes, we based the similarity score not on the pathogenic mutations but on genes carrying germline mutations or specific polymorphisms, which point to the uniqueness of the patient. All the non-pathogenic mutations correspond to irrelevant mutations that are part of the ccfDNA produced by normal cells.

The overall patient matching process is described in Figure 3C, in which the final output has similarity scores for all the liquid biopsy reports, and the best reports (scores closer to 1) are grouped together. K-means clustering, which uses most of the available irrelevant/non-tumorigenic gene mutations (including germlines, polymorphisms, and synonymous mutations), was performed to split all samples into two clusters or split the liquid biopsy results into groups that were more similar to one another. The cosine similarity analysis was then run for each file in comparison to the others in the bucket. If no match was identified for a patient report, it was rerun against the others. Lastly, the algorithm provided an output that indicated which files were most similar to one another, with these groups being essentially patients. The number of clusters (K = 2 in this study) was chosen based on the sample set available and could be changed when the sample size increased. 

The testing and confirmation indicated that the cosine similarity technique was effective in identifying similar reports by quantifying their similarity. The empirical cut-off identification was based on a manual test set of 10% of the samples for which we knew belonged to the same patient. For 56 genes and 60 samples, our score of 0.98 yielded 0 false positives. When we used this threshold on the rest of the unknown samples, the theoretical likelihood of incorrect matches was based on the likelihood of having twins in the sample set or the likelihood of missing critical germline mutations/polymorphisms in the genetic sequencing process. For the 600 samples considered, we reached a threshold score of 0.98 above which the likelihood of incorrect matching approaches 0 (no false positives). Scores over 0.98 belonged to the same patient in 100% of the cases based on external validation. Due to the presence of noise and inaccuracy in the sequencing of ctDNAs from the blood, some reports that belonged to the same patient could have scores less than 0.98. We have identified a score range, between 0.95 and 0.98, which contained reports that had the potential to belong to the same patient, i.e., a range that could have a potential match but not with 100% accuracy. An example of a result set is shown in Figure 3D. In some cases, these patients might be closely related to each other, having a high similarity in their genome, while some correspond to the same patients, but the inaccuracy stems out of sequencing errors. Even though, in a few cases, as described above, it could miss reports that belonged to the same patient, overall, our model performed well with no false positive matches. 

### 3.3. Longitudinal Visualization

**Figure 4:**
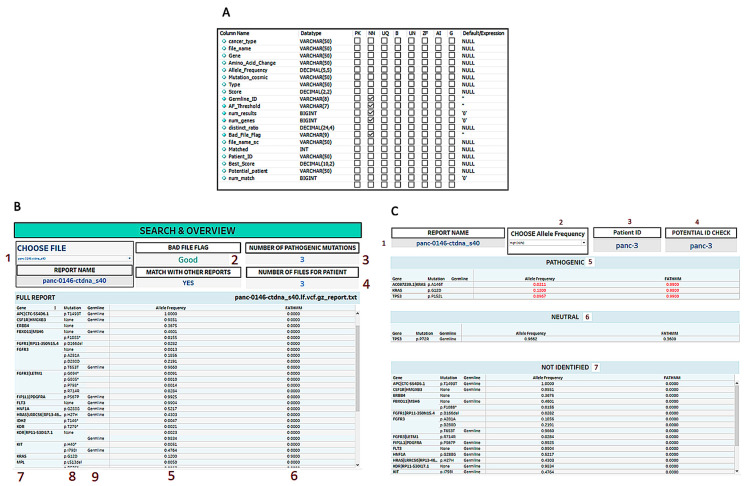
Longitudinal Visualization (**A**) MySQL Table Schema for the visualized Tableau output data. (**B**) Search & Overview page on the Tableau visualization. The following describes the elements in the Tableau interface: 1. Sample Name Selection: User can choose the sample based on the names available in the dataset; 2. Sample Sequencing Output Quality Check: A measure to determine the quality of the sequencing based on pre-identified factors that contribute to poor quality sequencing; 3. Pathogenic Mutations in the Sample: The number of pathogenic mutations that are seen in the sample based on our gene matching methodology; 4. Similar Samples: The number of similar samples in the database that are likely from the same patient based on our patient matching methodology; 5. Allele Frequency: Summarization of the allele frequency of gene mutations as per the report; 6. FATHMM Score: The pathogenicity indicator on a scale from 0 to 1, as reported by the COSMIC dataset hosted by the Sanger Institute, UK; 7. Genes: The name of the genes, as per the report; 8. Mutations: The identity of the mutations, as per the report; 9. Germline Mutations: The indication as to whether the gene mutation is germline. (**C**) Report page on the Tableau visualization. The following describes the elements in the Tableau interface: 1. Report Name: The indication of the report that is being reported in the page; 2. Allele Frequency Filter: The function used to filter mutations above 1% variant allele frequency; 3. Predicted Patient Matches: The patient name provided if the methodology clearly identifies a patient match based on previous available reports in the database; 4. Potential ID Check: A summary of the other potential patient matches if they exist. Otherwise, it shows the predicted match; 5. Pathogenic List: A list of identified pathogenic mutations in the report; 6. Neutral List: A list of identified mutations that are not clearly pathogenic; 7. Lower List: A list of identified mutations with a very low pathogenicity score.

Final results from the pathogenic matching and the patient similarity analysis were uploaded via the same Python script into an output database as structured in Figure 4A. Tableau software integrated this database and provided the dashboards shown in Figure 4B,C. We built the longitudinal analysis by combining the gene classification with the patient matching. Once we determined which anonymous liquid biopsies belonged to the same patient, it was possible to check how each pathogenic gene mutation evolved over time. From an efficiency perspective, this monitoring could be helpful as an indicator of the effectiveness of specific treatments and to spot additional cancer signals that would require further testing and clinical follow-up. Time is a crucial factor in cancer care. The opportunity to monitor the genes more closely and to be able to respond quickly can be lifesaving. 

**Figure 5:**
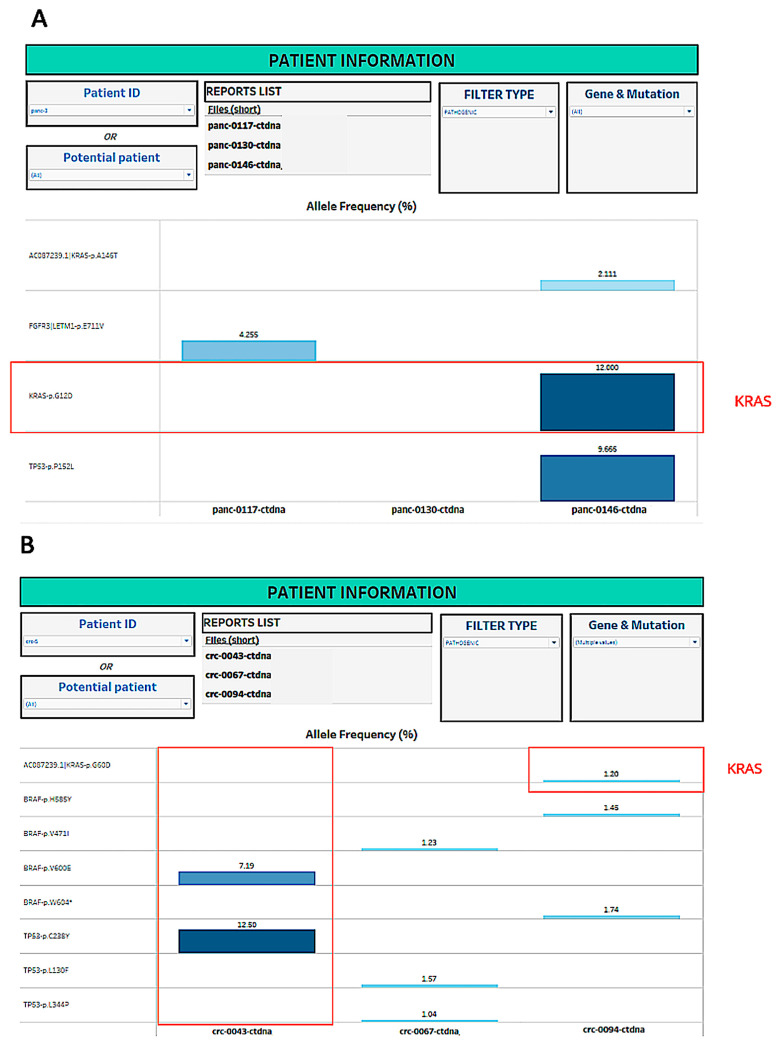
Longitudinal Visualization—case studies based on longitudinal analysis. (**A**) Pancreatic Cancer Patient—A case study based on longitudinal analysis. (**B**) Colorectal Cancer Patient—A case study based on longitudinal analysis.

Figure 5A,B provide an overview of two case studies used for longitudinal patient monitoring. Figure 5A shows the ongoing monitoring of a single pancreatic cancer patient that indicates the detection of a KRAS mutation in the third longitudinal blood draw. Figure 5B shows an overview of a second patient with colorectal cancer. A BRAF mutation was detected in the first blood draw, a subsequent sample indicated remission, and then a new pathogenic mutation in KRAS was detected in a third blood draw, suggesting cancer recurrence, which could prompt clinical staff for further follow-up.

## 4. Discussion

ctDNA analysis in peripheral blood is a liquid biopsy that contains representative tumor information, which includes information concerning gene mutations found in primary tumors [5]. These specific genetic changes found in ctDNA can have diagnostic value and can predict responses to treatment and patient survival. Additionally, as these liquid biopsies are easily obtainable, repeated samples can be taken for the real-time monitoring of both cancer patients’ response during the course of treatment and disease progression over time. As such, peripheral blood liquid biopsies that contain tumor-representative ctDNA have been proposed as an alternative to solid tumor biopsies [12].

The longitudinal visualization of the pathogenic gene mutations can improve patient monitoring [3]. With a limited test sample set, a way to address the theoretical likelihood of incorrect matches is to build a confidence interval of the empirical cut-off by randomly sampling the test set and applying the proposed methodology to identify the probabilistic occurrence of the score that makes the false positives equal to zero. Overall, this longitudinal visualization offers a non-invasive alternative to checking how the pathogenic genes are evolving. It can be used for the following strategies. First, it can help assess the effectiveness of a drug in treating the patient’s cancer. By monitoring the evolution of a known pathogenic mutation, the physician can measure the effect of a specific treatment in dealing with a particular kind of cancer. This is the principle of precision medicine [1]: to use information about a person’s gene abnormalities to use targeted treatment. Second, it can help identify new pathogenic gene mutations, which could be the consequence of clonal expansion within a heterogeneous tumor. Cancer is a disease in which tumor cells proliferate rapidly and, over time, can spread to other parts of the body. By monitoring the liquid biopsy reports, it might be possible to identify pathogenic mutations at an earlier stage, when the treatment is easier and more effective. 

The methodology presented here would help any team in a research context to build a data platform to automatically map identified mutations with their pathogenicity score and link reports to the same patient based on their genetic identity, i.e., their germline mutations, polymorphisms, and synonymous mutations originating from normal tissues. With improved technology, it might be possible to detect fragments of RNAs and/or proteins in the blood that might serve as a hint to signal disease or disorder in certain parts of the human body. Blood can serve as an important medium of inspection due to the presence of cell components circulating throughout the body [13]. The potential of detecting such fragments of DNA/RNA/protein could help identify and track the health of a patient over time. With the use of such data platforms, it would then be possible to evaluate many approaches for tracking patient health by using cellular fragments present in the blood to detect and/or monitor a variety of diseases including cancer. 

## Data Availability

The authors confirm that the data supporting the findings of this study are available within the article.
